# Formula

**Published:** 1852-05

**Authors:** 


*Formula.*

We have been requested to publish the formula for the preparation of Chloroform. It has already appeared in the *Journal*, but as some of our readers may not have access to the vol. containing it, we insert it again:

CHLOROFORM.

"Take of Chlorinated Lime ten pounds;
Water three gallons and a half;
Alcohol two pints.

"Mix the Chlorinated Lime first with the water, and then with the Alcohol, in a distillatory vessel having the capacity of about six gallons. Distil with a brisk heat into a refrigerated receiver, and, when the temperature approaches to 176°, withdraw the fire, in order that the distillation may proceed by the heat derived solely from the reaction of the materials. When the distillation slackens, hasten it by a fresh application of heat, and continue to distil until the liquid ceases to come over with a sweet taste. Separate the heavier layer of liquid in the receiver from the lighter by decantation, and, having washed it first with water, and then with a weak solution of carbonate of soda, agitate it thoroughly with powdered chloride of calcium, and distil it off by means of a water-bath, stopping the distillation when eleven-twelfths of the liquid have come over. The residue, together with the light liquid of the first distillation, may be reserved for use in a second operation.

"Chloroform is a colorless liquid, volatile, not inflammable, of a bland ethereal odor, and hot, aromatic, saccharine taste. Its specific gravity is 1.49, and boiling point 142°. It is slightly soluble in water, but freely so in alcohol and in ether. Mixed with an equal volume of sulphuric acid, it does not assume a reddish-brown color, nor is the acid discolored. When dropped into a cold mixture of equal weights of sulphuric acid and water, it sinks to the bottom. If a small quantity be added to distilled water, it forms transparent globules under the water, without assuming a milky appearance."

The accidents recently reported in the use of this article, and the impurities which it often contains, should admonish physicians and surgeons to be cautious both in its selection and administration. J.

